# Draft genome sequence and annotation of fungal pathogen *Cladosporium tenuissimum* SHK1100 causing postharvest sooty spot of fig fruit

**DOI:** 10.1186/s12863-026-01426-6

**Published:** 2026-04-21

**Authors:** Masahito Nakano

**Affiliations:** Toyo Institute of Food Technology, 4-23-2 Minami-Hanayashiki, Kawanishi-shi, Hyogo 666-0026 Japan

**Keywords:** *Cladosporium tenuissimum*, *Ficus carica*, Fig fruit, Genome assembly, PacBio sequencing, Postharvest, Sooty spot

## Abstract

**Objectives:**

*Cladosporium tenuissimum*, a widespread filamentous fungus belonging to the *C. cladosporioides* species complex, has been identified as a causal agent of sooty spot disease on postharvest fig fruits. Despite its ecological and agricultural importance, genomic resources for this species remain limited. Here, I report a draft genome sequence of *C. tenuissimum* strain SHK1100, isolated from symptomatic fig fruits in Japan, to facilitate comparative and functional genomic studies.

**Data description:**

The draft genome was assembled using long-read sequencing data, resulting in a total assembly length of 32.8 Mb, composed of 58 contigs with an N50 of 1.92 Mb and L50 of 8. The genome completeness of the assembly reached 98.0% in Benchmarking Universal Single-Copy Orthologs analysis with no contamination. In total, 12,016 protein-coding genes were predicted in the assembled genome. Functional annotation assigned putative functions to a large proportion of predicted genes, including annotations for 77.1% of genes into Clusters of Orthologous Groups, 33.7% of genes using Gene Ontology, and 21.6% of genes as putative effectors. This draft genome provides a foundation for understanding the pathogenic mechanisms and secondary metabolite biosynthesis of *Cladosporium* species associated with plant diseases.

## Objective

*Cladosporium*, a monophyletic genus within the *Cladosporiaceae* family, is recognized as one of the most abundant fungi in indoor and outdoor environments [1]. The genus is divided into three major species complexes—*C. herbarum*, *C. sphaerospermum*, and *C. cladosporioides*—based on their morphological features and multilocus DNA sequences. Members of this genus include species of ecological and industrial importance, as well as potential allergenic species and plant pathogens. *Cladosporium* can produce a wide range of secondary metabolites that exhibit biological activities, such as cytotoxicity [2], antimicrobial activity [3], and enzyme inhibition [4].

*C. tenuissimum*, a member of the *C. cladosporioides* species complex, is a widely distributed filamentous fungus frequently isolated from indoor environments, soil, and plant surfaces [1]. *C. tenuissimum* has been reported to cause disease on economically important crops, such as peach [5] and citrus [6]. In a previous study, *C. tenuissimum* was identified as the causal agent of sooty spot disease on postharvest fig (*Ficus carica*) fruits [7]. The fungus was isolated from symptomatic fruits collected from commercial markets and an experimental orchard of the Toyo Institute of Food Technology (34.81926°N, 135.40315°E) in Japan. Among the isolates, strain SHK1100 (MAFF248147) was characterized based on morphological features and multilocus sequence analysis using ITS, TEF1, and ACT [7].

Recent genome and transcriptome analyses of *Cladosporium* species have demonstrated their practical utility by revealing gene clusters and enzyme repertoires associated with plant growth promotion and organic pollutant degradation [8–10]. Despite its widespread occurrence, genomic information for *C. tenuissimum* remains limited, hindering comparative and functional genomic studies within this genus. Here, I report the draft genome sequence of *C. tenuissimum* SHK1100 generated using long-read sequencing technology. This genetic resource provides a foundation for further studies on the pathogenic mechanisms and secondary metabolite biosynthesis of *Cladosporium* species associated with postharvest fruit disease.

## Data description

For genome sequencing, the *C. tenuissimum* SHK1100 was grown in 100 mL of potato dextrose broth containing 100 µg/mL chloramphenicol for three days at 28 °C with shaking at 150 rpm. Mycelia (100 mg) were harvested by gravity filtration using filter paper (Advantec, Tokyo, Japan) and frozen in liquid nitrogen [11]. Genomic DNA was extracted using the NucleoBond HMW DNA kit (Takara Bio, Shiga, Japan) and purified using DNA Clean Beads (MGI Tech, Shenzhen, China). The integrity of DNA was assessed using a Genomic DNA ScreenTape (Agilent Technologies, Santa Clara, CA, USA) on an automated electrophoresis system TapeStation 4150 (Agilent Technologies), and the concentration of DNA was measured using a Qubit Fluorometer (Thermo Fisher Scientific, Waltham, MA, USA).

A PCR-free DNA library was prepared using the SMRTbell Prep Kit 3.0 (Pacific Biosciences, Menlo Park, CA, USA) after fragmenting the genomic DNA to 10–25 kb using a Megaruptor 3 (Diagenode, Liège, Belgium). The library was sequenced on a Revio system (Pacific Biosciences) by Bioengineering Lab. Co., Ltd. (Kanagawa, Japan). Raw reads were processed with SMRT Link v13.1.0.221970 to remove overhang adapter sequences and obtain HiFi reads (Q > 20), yielding 198,849 reads with an N50 of 7,430 bp (Table 1, Data set 1) [12]. HiFi reads longer than 5,000 bp were selected using SeqKit v2.10.0 [13] and subsequently assembled *de novo* using Hifiasm v0.21.0-r686 [14], resulting in 58 contigs with an N50 of 1,923,577 bp (Table 1, Data file 1, Data set 2) [15, 16]. Telomeric repeats were detected at both ends of 16 contigs using tidk v0.2.7 [17]. Assembly quality was assessed using QUAST-LG v5.3.0 [18]. Genome completeness was evaluated using Benchmarking Universal Single-Copy Orthologs (BUSCO) v5.7.0 with the dothideomycetes_odb10 dataset [19]. Average nucleotide identity values calculated using FastANI v1.34 [20] showed that *C. tenuissimum* SHK1100 shared 96.23% identity with *C. tenuissimum* ACCC 37043 (GCA_046128905.1) and 88.38% identity with *C. cladosporioides* SYC63 (GCA_022457075.1).

For gene structure annotation, protein-coding genes were predicted using BRAKER v3.0.8 [21] based on GeneMark-ES/EP+ [22, 23] and AUGUSTUS v3.5.0 [24], incorporating fungal protein homology from OrthoDB v12 [25]. In total, 12,016 protein-coding genes were identified in the assembled genome (Table 1, Data file 1, Data set 3) [16, 26] and no contaminants were detected by OMArk v0.3.1 [27]. Non-coding genes, such as tRNA and rRNA, were predicted using tRNAscan-SE v2.0.12 [28] and Barrnap v0.9 [29]. Additionally, repeat family identification and modeling were conducted using RepeatModeler v2.0.7 [30] and RepeatMasker v4.2.1 (Table 1, Data set 4) [31].

For gene functional annotation, predicted protein-coding genes were annotated using eggNOG-mapper v2.1.12 [32] against orthology-based database eggNOG DB v5.0.2, including Clusters of Orthologous Groups (COG) [33], Gene Ontology (GO) [34], Kyoto Encyclopedia of Genes and Genomes (KEGG) [35], carbohydrate-active enzymes (CAZy) [36], and Pfam [37], with DIAMOND v2.1.12 [38]. Additionally, secreted proteins and putative effectors were predicted using SignalP v6.0 [39] and EffectorP v3.0 [40], respectively, identifying 1,209 secreted proteins and 2,600 putative effectors. A summary of functional annotation is presented in Data file 2 (Table 1) [41].

## Limitations

This study presents a draft genome of *C. tenuissimum* generated using PacBio long-read sequencing. As the genome analysis was conducted on a single isolate, the results could not be generalized to the entire species and should be interpreted as strain-specific. Although the assembly shows high contiguity compared with short-read-based assemblies, it has not yet achieved complete chromosome-level resolution. A complete mitochondrial genome could not be identified in the assembly. Additionally, gene structure and functional annotations were generated using automated bioinformatics pipelines and public databases without experimental evidence; therefore, these findings remain preliminary. Further improvements, including scaffolding using Hi-C data and the integration of transcriptomic or proteomic evidence, would facilitate the generation of a more accurate chromosome-level genome assembly. Comparative genomic analyses with other *Cladosporium* species would further enhance our understanding of gene function and evolutionary relationships.

**Table 1:** Overview of data files/data sets

Label	Name of data file/data set	File types (file extension)	Data repository and identifier (DOI or accession number)
Data file 1	Genome assembly and annotation summary of *Cladosporium tenuissimum* SHK1100	MS Word file (.docx)	Figshare, 10.6084/m9.figshare.30948959 [16]
Data file 2	Functional annotation of genes in *Cladosporium tenuissimum* SHK1100	MS Excel file (.xlsx)	Figshare, 10.6084/m9.figshare.30948908 [41]
Data set 1	Sequencing read of *Cladosporium tenuissimum* SHK1100	Fastq file (.fastq)	NCBI Sequence Read Archive, http://identifiers.org/insdc.sra:DRR700011 [12]
Data set 2	Genome assembly of *Cladosporium tenuissimum* SHK1100	Fasta file (.fasta)	NCBI Nucleotide, https://identifiers.org/nucleotide:BAAJPQ000000000 [15]
Data set 3	Predicted genes of *Cladosporium tenuissimum* SHK1100	Fasta file (.fasta)	Figshare, 10.6084/m9.figshare.30949160 [26]
Data set 4	Gene annotation of *Cladosporium tenuissimum* SHK1100	General Feature Format file (.gff3)	Figshare, 10.6084/m9.figshare.31437025 [31]

## Data Availability

The data described in this Data note can be freely and openly accessed on NCBI Sequence Read Archive under BioProject PRJDB35511. Please see Table 1 and references [12, 15, 16, 26, 31, 41] for details and links to the data.

## Abbreviations

BUSCO: Benchmarking Universal Single-Copy Orthologs
CAZy: Carbohydrate-active enzymes
COG: Clusters of Orthologous Groups
GO: Gene Ontology
KEGG: Kyoto Encyclopedia of Genes and Genomes
